# Challenges in Identifying Uncommon Clinical Isolates of Candida Species Using Conventional Phenotypic Methods: A Clinical Concern

**DOI:** 10.7759/cureus.94311

**Published:** 2025-10-10

**Authors:** Leimapokpam S Devi, Madhuri, Mukesh Sharma, Moumita Sardar

**Affiliations:** 1 Microbiology, Faculty of Medicine and Health Sciences, Shree Guru Gobind Singh Tricentenary (SGT) University, Gurugram, IND

**Keywords:** candida albicans, chromogenic medium, conventional phenotypic methods, non-albicans candida spp, vitek 2 compact system

## Abstract

Background

*Candida* species, particularly non-albicans *Candida* species, have emerged as a major cause of human infection, often exhibiting multidrug resistance due to either acquired or intrinsic resistance to antifungals. Therefore, accurate speciation of *Candida *isolates is crucial. However, despite reported misidentification issues with conventional methods, many mycology laboratories, including those in resource-poor healthcare facilities, still rely on them. This study compared various *Candida* speciation methods, including conventional phenotypic methods, chromogenic media, and the VITEK 2 compact system (bioMérieux, Marcy l'Etoile, France), and assessed the antifungal susceptibility patterns of clinical isolates at a rural tertiary care hospital.

Methodology

This prospective cross-sectional study was conducted from May 2024 to April 2025. *Candida* species were isolated from various clinical specimens (blood, urine, sputum, and pus) and subsequently speciated using conventional phenotypic methods (germ tube test, growth at 45°C, carbohydrate fermentation, and assimilation tests), HiCrome *Candida *differential agar, and the VITEK 2 compact system. Antifungal susceptibility testing was performed using the disc diffusion method on Mueller-Hinton agar supplemented with methylene blue.

Results

A total of 78 *Candida* isolates were obtained during the study period, with the majority isolated from urine (n = 44; 56.4%). Risk factor analysis of patients with *Candida* infections revealed that long-term antibiotic use (22.5%) was the most frequently associated factor. Among the isolates, seven were identified as *Candida albicans*, while the remaining isolates were non-albicans *Candida *species, including *Candida tropicalis* (n = 53), *Candida parapsilosis *(n = 15), *Candida auris* (n = 2), and *Candida glabrata* (n = 1). The VITEK 2 YST ID results were considered the reference method, and conventional phenotypic and chromogenic medium methods showed a varying degree of accuracy. Neither conventional phenotypic nor chromogenic medium accurately identified *C. auris*. High resistance rates were observed for fluconazole (57.7%), itraconazole (n = 66.7%), and voriconazole (60.3%), with non-albicans* Candida *species exhibiting higher resistance than *C. albicans*.

Conclusion

This study highlights the increasing trend of non-albicans *Candida* species as the predominant cause of *Candida* infections in rural Haryana, India, emphasizing the need for accurate *Candida* species identification due to their high multidrug resistance. Notably, conventional phenotypic methods and chromogenic media failed to accurately identify *C. auris*, highlighting the limitations of these approaches.

## Introduction

*Candida* species have emerged as a major cause of human infection over the past two decades, leading to high mortality rates [1-3]. While it is a commensal in the mucosal oral cavity, gastrointestinal tract, and vagina in healthy humans, it is considered an opportunistic fungal pathogen causing superficial infections of skin and mucous membranes to systemic life-threatening invasive infections [1,3,4]. The high mortality rates associated with *Candida* infections could be due to the increasing incidence of invasive and septicemic infections, particularly among immunocompromised individuals [3]. Globally, *Candida* species have been recognized as a leading cause of hospital-acquired infections, with systemic *Candida* infections ranking fourth among the most common causes of bloodstream infections (BSIs) acquired in hospitals [3,5]. Among *Candida* species, *Candida albicans* is the most prevalent species globally, with the exception of a few Asian nations where non-albicans *Candida* (NAC), specifically *Candida tropicalis,* is the predominant species [6,7]. An Indian study revealed an alarming prevalence, with NAC isolates accounting for 63.3% of cases, and *C. tropicalis* being the most prevalent, followed by *Candida glabrata* and *Candida krusei* [8].

Despite the fact that clinical manifestations of infections associated with different members of NAC are usually indistinguishable, several NAC species either acquire resistance over time or have intrinsic resistance against commonly used antifungals [5]. Several virulence factors facilitate the transition of *Candida* species from commensal to a potent pathogen, including adherence to host tissues and medical devices, formation of biofilm, secretion of extracellular hydrolytic enzymes (e.g., proteases, lipases, phospholipases, esterases, and phosphatases), toxins, complement receptors, and phenotypic switching [9]. Therefore, identification of *Candida* isolates to the species level is essential for effective treatment of patients with *Candida* infections.

While several studies have reported the prevalence and distribution of *Candida* species in different clinical settings, there is a lack of information on their characterization and susceptibility patterns in rural tertiary care centers, where these facilities often face challenges, such as limited resources, lack of specialized expertise, and delayed access to diagnostic facilities. These factors can impact the timely identification and management of *Candida* infections, potentially leading to suboptimal treatment outcomes [10]. Moreover, conventional phenotypic methods for *Candida* speciation, which commonly use morphological and biochemical properties of each species, often misidentify uncommon *Candida* species such as *Candida kefyr*, *Candida auris*, and *Candida lusitaniae* [11]. Therefore, this study aimed to compare *Candida* speciation methods, including conventional phenotypic methods, growth on chromogenic medium, and the VITEK 2 compact system (bioMérieux, Marcy l'Etoile, France), among *Candida* species isolated from various clinical samples from patients attending a rural tertiary care hospital. Additionally, the antifungal susceptibility pattern of the isolates to widely used antifungal drugs was assessed.

## Materials and methods

This prospective cross-sectional study was conducted from May 2024 to April 2025 at the Microbiology Laboratory, Shree Guru Gobind Singh Tricentenary (SGT) Hospital, Haryana, India. The study protocol received approval from the Institutional Ethical Committee (IEC/FMHS/PhD/2023-05).

Study population

Inclusion Criteria

Patients of all ages and genders with clinically suspected candidiasis who provided written consent were included in the study. A pre-designed proforma, adapted from a CDC document [12], was used to collect information on age, gender, and other demographic characteristics, as well as risk factors such as diabetes, recent surgery, long-term antibiotic use, and use of indwelling medical devices, for further data analysis.

Exclusion Criteria

Patients receiving antifungal therapy were excluded to prevent potential interference with the antifungal susceptibility testing results.

Collection of *Candida* isolates

**Figure 1:**
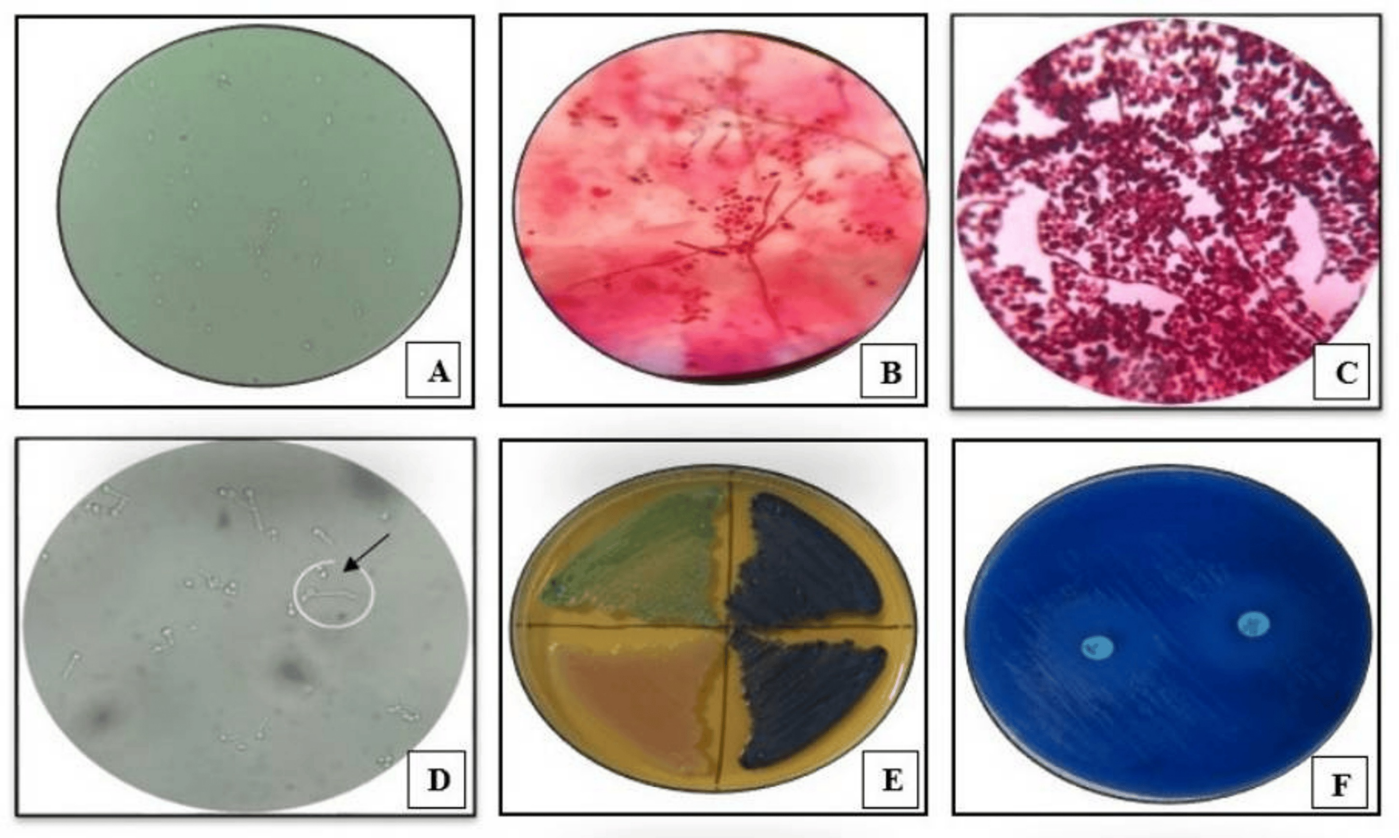
Identification and antifungal susceptibility testing of Candida species (A) Potassium hydroxide mount of sputum sample showing budding yeast cells (40x). (B) Gram stain of sputum sample showing Gram-positive budding yeast cells with pseudohyphae (100x). (C) Gram stain of fungal culture showing Gram-positive budding yeast cells with pseudohyphae (100x). (D) Germ tube test positive, wet mount preparation (40x). (E) HiCrome *Candida* differential agar showing differential growth of *Candida* species, *Candida albicans* (light green), *Candida tropicalis* (blue), and *Candida glabrata* (cream). (F) Antifungal susceptibility testing of *Candida* species on Mueller-Hinton agar supplemented with methylene blue

*Candida* species (n = 78) were isolated from various clinical specimens, including blood, sputum, pus, and urine from the study population by standard microbiological techniques. The isolates were subsequently included in the study for further speciation using standard conventional phenotypic methods, growth on chromogenic *Candida* differential medium (HiCrome *Candida *differential agar), and the VITEK 2 compact system. Isolation of *Candida* species was performed by subculturing on Sabouraud dextrose agar (SDA) supplemented with chloramphenicol. Blood cultures were processed using the BacT/Alert system (bioMérieux, Marcy l'Etoile, France), and after flagging positive, subcultures were done on SDA for *Candida* isolation. All inoculated SDA tubes were incubated at 37°C for 24-48 hours, and the isolates were further subjected to various methods for speciation and antifungal susceptibility testing (Figure 1).

Methods for speciation of *Candida* isolates

Conventional phenotypic methods for *Candida* speciation, such as the germ tube test, growth at 45°C, carbohydrate fermentation, and carbohydrate assimilation, were performed [13]. For the germ tube test, isolates showing characteristic germ tube formation within 2-3 hours of incubation in human serum at 37°C were considered positive. For growth at 45°C, isolates with positive germ tube test results were inoculated on SDA and incubated at 45°C for 48 hours; *C. albicans* grows at this temperature, helping to differentiate it from *Candida dubliniensis*, which is also a germ tube-positive *Candida* species. Carbohydrate fermentation was carried out using Andrade peptone water with individual carbohydrates (glucose, maltose, lactose, and sucrose), and acid and gas production were observed to determine the fermentative capability of the isolate. Carbohydrate assimilation testing involved assessing the ability of *Candida* isolates to utilize different carbohydrates as the sole carbon source, evaluated by growth in media containing individual carbohydrates.

HiCrome *Candida* differential agar (HiMedia Laboratories Pvt. Ltd., Mumbai, India) was used to differentiate *Candida* species based on colony color and morphology, with species-specific chromogenic reactions aiding in identification. Species-specific colony colors are as follows: light green colonies for *C. albicans*, cream to white for *C. glabrata*, metallic blue colonies for *C. tropicalis*, purple fuzzy colonies for *C. krusei*, and white to cream color colonies for *C. parapsilosis*. The VITEK 2 compact system is an automated system with fluorescence-based detection and colorimetric technology that employs a VITEK 2 YST ID card, utilizing 46 biochemical reactions for rapid and accurate yeast species, specifically for *Candida* isolates within 18-24 hours [14].

Antifungal susceptibility testing for all the *Candida* isolates was performed using the disc diffusion method and interpreted according to Clinical and Laboratory Standards Institute (CLSI) guidelines [15]. The following antifungal discs from HiMedia Laboratories Pvt. Ltd., India, were used: fluconazole (25 µg), itraconazole (10 µg), and voriconazole (1 µg). *C. albicans* ATCC 90028 and *C. tropicalis* ATCC 750 were used as quality control strains.

Statistical analysis

Data on categorical variables including antifungal susceptibility results were expressed as frequencies and percentages. Comparisons of conventional phenotypic methods, growth on chromogenic medium, and the VITEK 2 compact systems for speciation of *Candida *isolates were evaluated using the Chi-squared test. The association between risk factors with *Candida *infections was analyzed by using the odds ratio [16]. A p-value < 0.05 was considered statistically significant.

## Results

A total of 78 *Candida* species were isolated from various clinical specimens obtained from patients attending different clinical departments. The patients’ ages ranged from two to 79 years, with a mean age of 42.3 years. The majority of the patients were women (52.6%), while men accounted for 37 cases (47.4%). Among the 78 patients, 58 (74.4%) were from inpatient departments (IPD) and 20 (25.6%) were from outpatient departments (OPD) (Table 1).

**Table 1:** Demographic characteristics of the study population (n = 78) IPD: inpatient department; OPD: outpatient department

Variables		Description
Age (years)	Range	2-79
	Mean ± SD	42.3 ± 18.9
Gender	Male	37 (47.4%)
	Female	41 (52.6%)
	Male:female	1:1.1
Patients attending	IPD	58 (74.4%)
	OPD	20 (25.6%)

**Figure 2:**
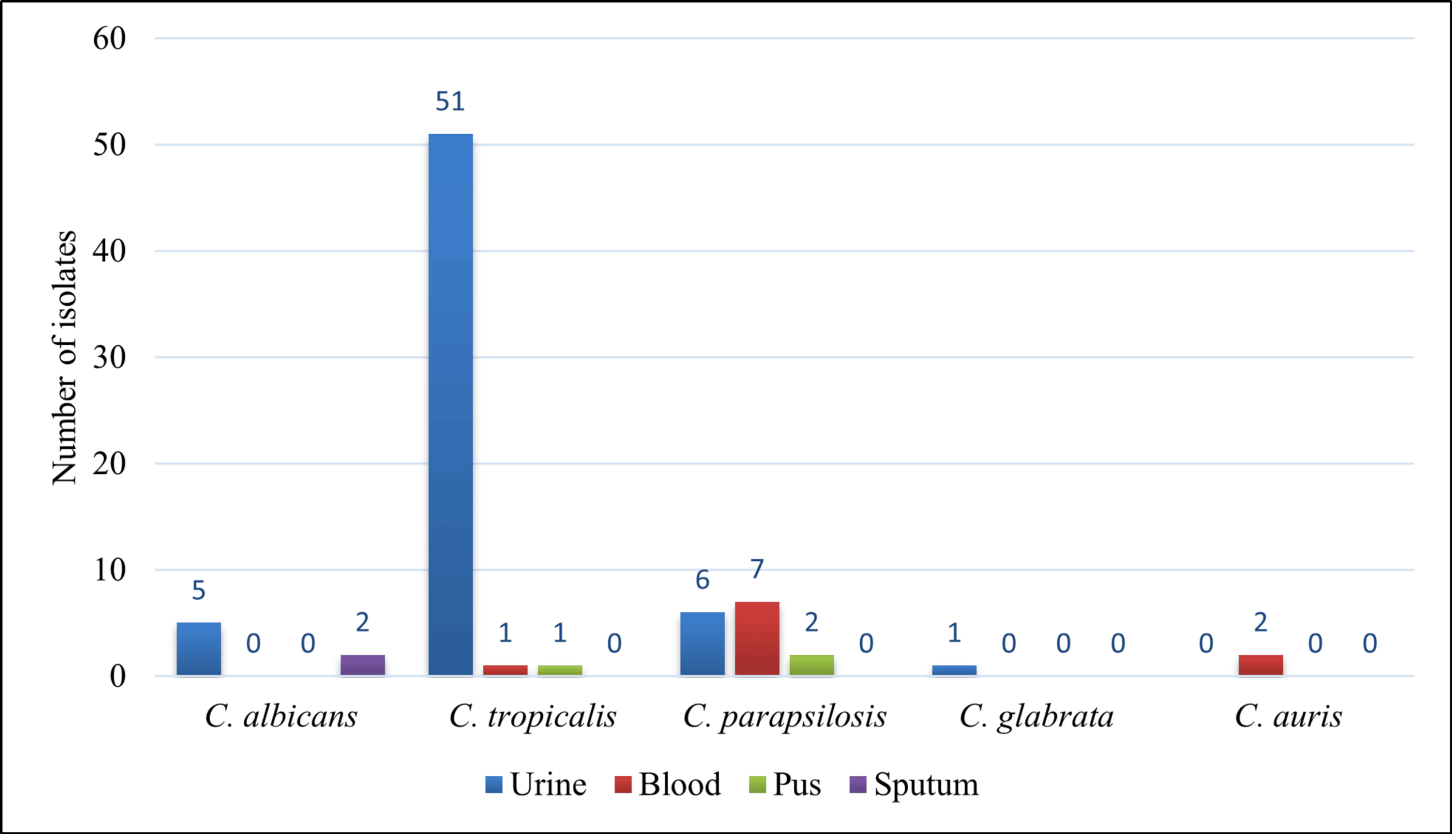
Distribution of Candida species among various clinical specimens

The distribution of *Candida* species by specimen revealed varied manifestations, with the majority isolated from urine (n = 44; 56.4%), followed by blood (n = 19; 24.4%), pus (n = 6; 7.7%), and sputum (n = 9; 11.5%). A total of seven *Candida* isolates were identified as *C. albicans*, while the remaining isolates were NAC species. The majority (five out of seven) of the *C. albicans* were from urine samples, and two were isolated from sputum samples (Figure 2). All *Candida* isolates from blood and pus were NAC species. Of the 53 *C. tropicalis* isolates identified, 51 (96.2%) were isolated from urine, while the other two isolates were obtained from blood and pus. Seven out of 15 *C. parapsilosis* isolates were obtained from blood, six from urine, and two from pus. All *C. auris* isolates were from blood, whereas a *C. glabrata* strain was obtained from urine. When the distribution of *Candida* species by specimen type and patient type was assessed, no statistical significance was observed (p > 0.05, calculated using the χ^2^ test).

Risk factor analysis of patients with *Candida* infections indicated that long-term antibiotic use (22.5%) followed by diabetes (16.7%) and prolonged intensive care unit (ICU) stays (14.1%) were frequently associated factors (Table 2). The odds of exposures, including diabetes, urinary catheterization, long-term antibiotic use, and prolonged ICU stays, were higher among patients with *C. albicans* infections than those with NAC species infections, highlighting a positive association of these risk factors and *C. albicans* infections. However, there was no statistically significant difference between the two types of infections in terms of chronic kidney disease, mechanical ventilation, and immunosuppression (p < 0.05).

**Table 2:** Assessment of risk factors associated with Candida infections NAC: non-albicans *Candida*; NS: not significant (p > 0.05, calculated using odds ratio)

Risk factors	Present in (total = 78) No. (%)	Type of *Candida* infections		Statistical analysis (odds ratio)		
		*Candida albicans* (n = 7) No. (%)	NAC (n = 71) No. (%)	Odds ratio	95% CI	p-value
Diabetes	13 (16.7)	5 (38.5)	8 (61.5)	19.68	3.26-118.78	0.001
Chronic kidney disease	6 (7.7)	2 (33.3)	4 (66.7)	6.7	0.97-45.9	NS (0.05)
Urinary catheterization	8 (10.3)	3 (37.5)	5 (62.5)	9.9	1.71-57.05	0.01
Mechanical ventilation	6 (7.7)	2 (33.3)	4 (66.7)	6.7	0.97-45.9	NS (0.05)
Long-term antibiotic use	16 (20.5)	6 (37.5)	10 (62.5)	36.6	3.97-337-03	0.001
Long periods in intensive care unit	11 (14.1)	4 (36.4)	7 (63.6)	12.1	2.25-65.94	0.003
Immunosuppression	4 (5.1)	1 (25)	3 (75)	3.7	0.33-42.15	NS (0.28)

Comparison of various *Candida* speciation methods for accuracy

The identification of *Candida* species was performed using standard conventional tests, including the germ tube test, growth at 45°C, carbohydrate fermentation, and assimilation tests. Out of the 78 clinical isolates, seven (9%) tested positive in the germ tube test, confirming their identification as *C. albicans*/*C. dubliniensis*, while the remaining 71 (91%) germ tube-negative isolates were classified as NAC. Furthermore, growth at 45°C was observed in all the germ tube-positive *Candida* isolates. Patterns of carbohydrate fermentation and assimilation by the *Candida* isolates revealed that seven of the isolates were those of *C. albicans*. The remaining NAC species included 53 isolates of *C. tropicalis*, 17 isolates of *C. parapsilosis*, and one isolate of *C. glabrata* (Table 3). *C. albicans* fermented glucose and maltose with acid and gas production, while it assimilated glucose, sucrose, and trehalose. *C. tropicalis* fermented glucose, sucrose, and maltose with acid and gas production, while it assimilated glucose, sucrose, and trehalose. *C. parapsilosis* fermented glucose while it assimilated glucose, sucrose, and trehalose. *C. glabrata* fermented glucose, whereas it assimilated both glucose and trehalose.

**Table 3:** Carbohydrate fermentation and assimilation pattern of Candida species (n = 78) F: fermented with acid & gas production; A: carbohydrate assimilation-positive; -: not fermented or carbohydrate assimilation-negative

*Candida* species, n = 78	Carbohydrate fermentation test				Carbohydrate assimilation test				
	Glucose	Sucrose	Maltose	Lactose	Glucose	Sucrose	Lactose	Trehalose	Raffinose
*C. albicans* n = 07 (9%)	F	-	F	-	A	A	-	A	-
*C. tropicalis* n = 53 (68%)	F	F	F	-	A	A	-	A	-
*C. parapsilosis *n = 17 (21.8%)	F	-	-	-	A	A	-	A	-
*C. glabrata* n = 01 (1.2%)	F	-	-	-	A	-	-	A	-

Based on colony color and morphology on HiCrome *Candida* differential agar, the majority (52/78) of the *Candida* isolates were identified as *C. tropicalis*, followed by *C. parapsilosis* (16/78) and *C. albicans* (7/78). Three *Candida* isolates could not be identified due to non-specific colony color and morphology on the chromogenic medium (Table 4).

**Table 4:** Comparison of phenotypic methods for speciation of Candida species (n = 78) Sensitivity and specificity were calculated considering the results of the VITEK 2 compact system as the reference method ^*^Two *C. auris* could not be identified by conventional methods and HiCrome *Candida* differential agar ^¥^One *C. tropicalis* could not be identified due to non-specific colony color on HiCrome *Candida* differential agar ^€^One *C. glabrata* was misidentified as *C. parapsilosis* due to similar colony color on HiCrome *Candida* differential agar

*Candida* spp.	Conventional methods			HiCrome *Candida* differential agar			VITEK 2 YST ID No. (%)
	No. (%)	Sensitivity (%)	Specificity (%)	No. (%)	Sensitivity (%)	Specificity (%)	
C. albicans	7 (9)	100	100	7 (9)	100	100	7 (9)
C. tropicalis	53 (68)	100	100	52 (66.7)^¥^	98.1	100	53 (68)
C. parapsilosis	17 (21.8)	100	96.9	16 (20.5)^€^	100	98.4	15 (19.2)
C. glabrata	1 (1.2)	100	100	0 (0)	0	0	1 (1.2)
C. auris	0 (0)^*^	0	0	0 (0)^*^	0	0	2 (2.6)
Total	78 (100)	-	-	75 (96.2)	-	-	78 (100)

All *Candida* isolates were subjected to an automated identification system using the VITEK 2 YST ID, which was considered the reference method due to its ability to identify uncommon *Candida* species. The most frequently isolated *Candida* species was *C. tropicalis* (53/78), followed by *C. parapsilosis* (15/78), *C. albicans* (7/78), *C. auris* (2/78), and *C. glabrata* (1/78) (Table 4).

The comparison of results from different phenotypic methods used to speciate the 78 *Candida* species is shown in Table 4. The VITEK 2 YST ID was considered the reference method for speciation, and subsequent comparisons with other methods were conducted. For the identification of *C. albicans*, *C. tropicalis*, and *C. glabrata*, the agreement between conventional methods and the VITEK 2 YST ID was 100%. For the identification of *C. parapsilosis*, the sensitivity and specificity of conventional methods were found to be 100% and 96.9%, respectively. Although *Candida* species may be rapidly and cost-effectively identified using HiCrome *Candida* differential agar, non-specific or atypical colony color or morphology may result in misidentification. The specificity for identifying *C. tropicalis* using chromogenic medium was 100%, while the sensitivity was 98.1%. For the identification of *C. parapsilosis*, the sensitivity and specificity were 100% and 98.4%, respectively. Both conventional methods and chromogenic medium misidentified or failed to identify *C. auris* strains. These findings emphasize the utility of combined phenotypic methods for preliminary identification and highlight the importance of automated systems like the VITEK 2 YST ID for accurate identification, especially of phenotypically similar or emerging *Candida* species.

Antifungal resistance profile

Overall, the antifungal resistance profile of *Candida* isolates was 57.7% for fluconazole, 66.7% for itraconazole, and 60.3% for voriconazole (Table 5). Although the number of *C. albicans* isolates was lower compared to NAC species, their antifungal resistance profiles were analyzed. NAC species showed higher resistance to all antifungals tested compared to *C. albicans*, although the difference was statistically insignificant. The two *C. auris* isolates and one *C. glabrata* isolate exhibited resistance to all antifungals, i.e., fluconazole, itraconazole, and voriconazole. *C. parapsilosis* also showed higher resistance to the antifungals (53.3% to 80%).

**Table 5:** Antifungal resistance pattern of C. albicans and non-albicans Candida (n = 78)

Antifungal agents	Antifungal resistance						
	Total (n = 78) No. (%)	*C. albicans* (n = 7) No. (%)	Non-albicans *Candida*				
			Total (n = 71) No. (%)	*C. tropicalis* (n = 53) No. (%)	*C. parapsilosis* (n = 15) No. (%)	*C. auris* (n = 2) No. (%)	*C. glabrata* (n = 1) No. (%)
Fluconazole	45 (57.7)	3 (42.9)	42 (59.2)	27 (50.9)	12 (80)	2 (100)	1 (100)
Itraconazole	52 (66.7)	3 (42.9)	49 (69)	34 (64.2)	12 (80)	2 (100)	1 (100)
Voriconazole	47 (60.3)	3 (42.9)	44 (62)	33 (62.3)	8 (53.3)	2 (100)	1 (100)

## Discussion

Fungi, previously deemed non-pathogenic or less virulent, are now acknowledged as a primary contributor to morbidity and mortality in immunocompromised and critically ill patients [9]. The incidence of *Candida* infections, mostly opportunistic infections in immunocompromised individuals, has been increasing globally [3]. In immunocompromised individuals, *Candida* species can cause invasive infections that may disseminate to internal organs. Furthermore, new treatments for various diseases, surgery, long-term ICU stays, and earlier broad-spectrum antibiotic usage have increased the number of immunosuppressed individuals, thereby increasing the incidence of disseminated candidiasis [2,3,17].

**Figure 3:**
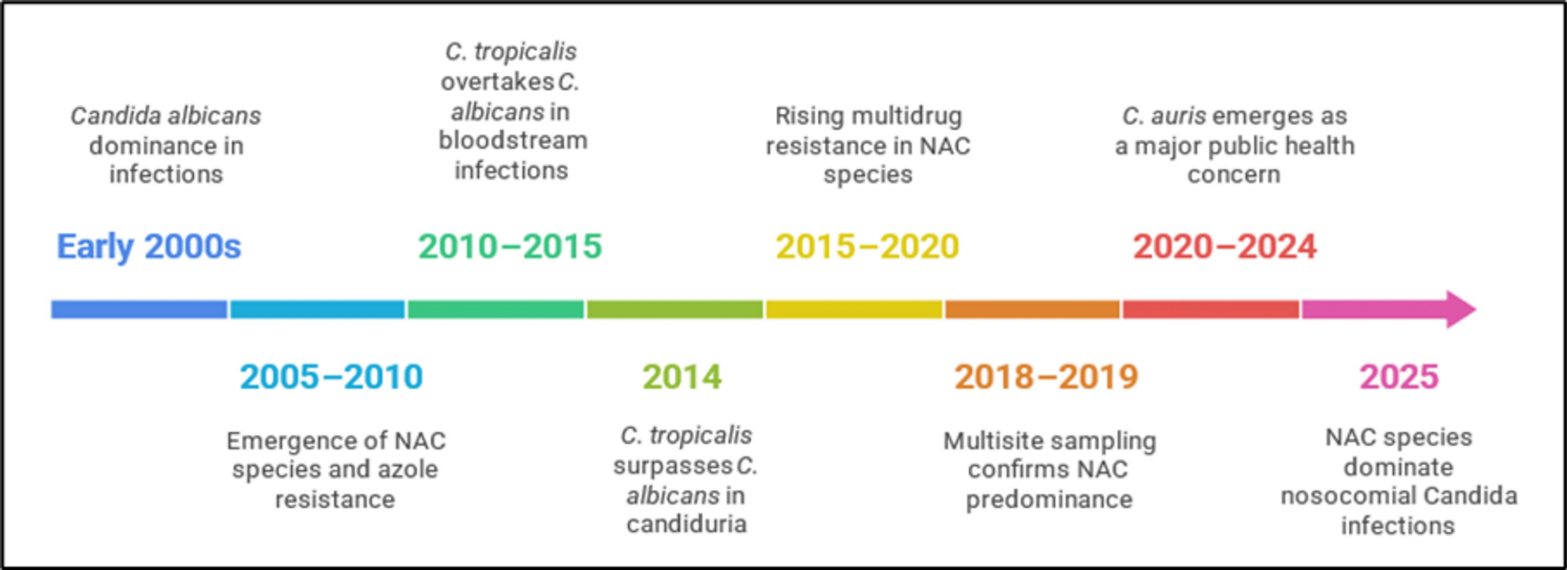
Evolution of Candida species in clinical settings in India Image credits: Sardar M generated this image using Napkin AI tool (accessed on August 9, 2025), using findings from references [19,21-27]

Globally, *C. albicans* was once the predominant etiological agent causing candidiasis. However, a recent trend toward NAC species has been reported [7,8]. A comparable pattern has been observed in the present study, highlighting a notable shift toward an increasing incidence of *Candida* infections due to NAC species, such as *C. tropicalis*, *C. parapsilosis*, *C. auris*, and *C. glabrata*. In contrast to earlier studies conducted at the same hospital, which reported incidence rates of *C. albicans* infections as 51.6% (years 2011-2013) [18] and 40% (years 2016-2019) [19], the present study shows a significant decline in *C. albicans* infections to 9%, indicating a changing epidemiology of *Candida* infections. Among NAC species, *C. tropicalis* is often associated with nosocomial infections and has been reported as the second most commonly isolated species after *C. albicans* [20]. This is corroborated by the present study, which identified *C. tropicalis* as the most frequently isolated NAC species (74.6%, 53/71). A timeline for the evolution of clinical isolates of *Candida* species in Indian healthcare settings is depicted in Figure 3. This finding is consistent with the significant shift reported by Deorukhkar et al. from Western India, who found that 63.3% (331/523) of *Candida* species isolated from various clinical specimens were NAC species, with *C. tropicalis* (35.1%, 116/331) being the most predominant species, followed by *C. glabrata* (28.1%) and *C. krusei* (16.3%) [8]. Furthermore, Ahmed et al. from Northern India reported a higher frequency of NAC species (85.9%, 61/71) among *Candida* isolates from blood cultures, with *C. tropicalis* (35.2%) as the most common species, followed by *C. glabrata* (21.1%) and *C. krusei* (9.9%). Additionally, rare *Candida* species such as *Candida guilliermondii*, *Candida pelliculosa*, and *Candida utilis* have been reported [21]. BSIs caused by *C. auris* are associated with high mortality rates of 30%-60% [22]. In the present study, all *Candida* isolates from blood cultures were identified as NAC species, including two *C. auris* strains, and were obtained from hospitalized patients. A study by Rajni et al. conducted in ICUs of two hospitals in Northwestern India reported *C. auris* as the predominant *Candida* species, constituting 42% of candidemia cases, which is consistent with other studies showing *C. auris* as an emerging cause of concern in Indian healthcare settings [23].

The increasing prevalence of infections caused by NAC species has been reported, accounting for more than 50% of invasive candidiasis in a sentinel-surveillance study in the United States and Canada [2]. In the present study, although the majority of NAC species were isolated from urine (81.7%), over half of the *C. parapsilosis* and all *C. auris* isolates originated from candidemia patients. The significant incidence of urinary tract infections attributed to *Candida* species highlights the emergence of this fungus as a notable etiology, particularly in IPD patients (70%, n = 44). Similar to our findings, Deorukhkar et al. reported that the majority of *Candida* isolates were from urine, with 60.8% identified as NAC species and 39.2% as *C. albicans* [8]. In addition to being better adapted to the urinary system, NAC species are more challenging to eliminate than *C. albicans* [7]. Risk factors such as the presence of urinary catheters, constant exposure to antifungal drugs, urinary abnormalities, advanced age, diabetes mellitus, abdominal surgeries, and pregnancy are frequently associated with candiduria [24]. Overall risk factor analysis of patients in the present study revealed prolonged antibiotic usage, diabetes, and extended ICU stays as frequently associated factors. Aruna and Jahappriya found diabetes mellitus to be the most frequently associated risk factor among patients with various *Candida* infections [25]. Another study reported an increased risk of acquiring nosocomial *Candida* BSIs in severe COVID-19 patients who had prolonged ICU stays, multiple courses of broad-spectrum antibiotics, mechanical ventilation, and other invasive devices [23].

Phenotypic methods are predominantly used for diagnosing fungal infections in Asia, with 81.7% of laboratories depending on non-commercial phenotypic methods and 44.7% also employing commercial phenotypic methods used for identifying *Candida* species [11]. The present study evaluated the accuracy of various phenotypic methods employed for the identification of *Candida* species. Phenotypic methods, including conventional methods such as the germ tube test, growth at 45°C, carbohydrate fermentation and assimilation tests, and growth on HiCrome *Candida* differential agar, were compared with the results obtained through the automated VITEK 2 compact system. The VITEK 2 YST ID identified two *C. auris* strains among the *Candida* isolates, whereas conventional methods erroneously identified both strains as *C. parapsilosis*. Additionally, growth on HiCrome *Candida* differential agar resulted in the inability to identify two *C. auris* strains, one *C. tropicalis*, and one *C. glabrata* due to non-specific colony color on the medium. A high rate of misidentification of uncommon *Candida* species by conventional methods has been reported [11]. The present study found 100% sensitivity for phenotypic conventional methods in identifying *Candida* species, including the germ tube test, growth at 45°C, and carbohydrate fermentation and carbohydrate assimilation tests. The specificity for identifying *C. parapsilosis* was 96.9%, attributed to the misidentification of two *C. auris* strains as *C. parapsilosis*. Although phenotypic conventional methods are considered the gold standard for identifying *Candida* species, they are labor-intensive and time-consuming. Additionally, it has been reported that there is no reliable phenotypic conventional method to accurately distinguish between closely related germ tube test-positive *Candida* species, often leading to misidentification of *C. dubliniensis* and *Candida africana* strains as *C. albicans* in clinical mycology laboratories [26]. Moreover, recently identified uncommon *Candida* species, e.g., *C. auris*, may be misidentified as several other organisms when using conventional or traditional phenotypic methods for yeast identification [27]. In the present study, all the *C. albicans* strains were accurately identified by HiCrome *Candida* differential agar; however, the sensitivity and specificity for identifying NAC species were 87.2% and 98.6%, respectively. Mutlu Sariguzel et al. evaluated the performance of CHROMagar *Candida*, VITEK 2 YST, and VITEK MS for identifying *Candida* isolates. In their study, two *Candida* isolates were misclassified as *C. parapsilosis* by CHROMagar *Candida*, and the speciation of *C. glabrata* was inconsistent with the reference method [28]. Saxena et al. reported that the VITEK 2 system accurately differentiated closely related *Candida* species, including uncommon species of *Candida* such as *C. albicans*, *Candida famata*, *Candida ciferrii*, *Candida gulleri*, and *C. tropicalis*, using 15 h using the VITEK 2 system, in contrast to identification by growth on chromogenic medium [29]. According to Esmat et al., the VITEK 2 demonstrated a sensitivity of 96% and specificity of 100% in accurately identifying *Candida* species, outperforming CHROMagar, which showed a sensitivity of 89% and specificity of 100% [30].

NAC species infections have clinical manifestations similar to those of *C. albicans* infections; however, they differ in terms of epidemiology, virulence factors, and especially the pattern of resistance to antifungal drugs [2,7,8]. Multidrug-resistant strains including *C. auris* have been reported among NAC species due to their ability to evade the mechanisms of action of different antifungal drugs, thus developing resistance. Additionally, some of these *Candida* species have demonstrated the ability to evade host immunity. The present study revealed that NAC species exhibited higher resistance to fluconazole and itraconazole, while *C. albicans* showed slightly higher resistance to voriconazole. This resistance to azoles may be attributed to alterations in target enzymes, limited drug access to the target, or a combination of both. Consequently, the significant resistance to azoles, which are the primary treatment choices, poses significant challenges in clinical practice. Our findings are in concordance with those of Deorukhkar et al. and Aruna and Jahappriya, which revealed that NAC species exhibited higher resistance to azoles than *C. albicans* in isolates from diverse specimens [8,25]. These findings suggest that the widespread or injudicious use of azoles has contributed to the development of resistance to these commonly relied upon antifungal drugs. Accurate identification of NAC species, especially *C. auris*, is therefore essential for effective treatment and infection prevention.

The limitations of this prospective cross-sectional study include a relatively small sample size and the restricted carbohydrate panel used in fermentation and assimilation tests for *Candida* species identification. Another limitation was its focus solely on azoles, whereas a more comprehensive approach would have included other antifungal classes, such as polyenes (e.g., amphotericin B) and pyrimidines (e.g., flucytosine), as well as newer antifungal drugs, e.g., caspofungins. Furthermore, determining the minimum inhibitory concentration for each antifungal drug by *C. albicans* and NAC species would have provided more nuanced and clinically relevant results, enabling healthcare providers to make more informed treatment decisions and optimize patient care.

## Conclusions

The present study highlights an increasing trend of NAC species as the predominant cause of *Candida* infections among patients in rural Haryana, India. This worrisome emergence of NAC spp., almost replacing the commonly isolated species *C. albicans* in rural communities, deserves attention to promote accurate identification of *Candida* species and regular antimicrobial surveillance, which is necessary to monitor changes in antifungal resistance patterns.

Accurate identification of clinical *Candida* isolates is crucial for choosing the right antifungal treatment, as NAC species often show increased resistance to antifungals. Although rapid and reliable automated systems, such as the VITEK 2 compact system, and molecular diagnostic methods are available, their high cost restricts widespread use, making conventional techniques the primary means of identifying *Candida* species in most clinical laboratories, including those in developing countries. However, misidentification of rare *Candida* spp. such as *C. auris* by the conventional method often occurs.
